# A CROSSWALK OF COMMONLY USED FRAILTY SCALES

**DOI:** 10.1093/geroni/igac059.2995

**Published:** 2022-12-20

**Authors:** Stephanie Denise Sison, Sandra Shi, Kyung Moo Kim, Gahee Oh, Nessa Steinberg, Sohyun Jeong, Ellen McCarthy, Dae Kim

**Affiliations:** Beth Israel Deaconess Medical Center, Brookline, Massachusetts, United States; Hebrew Senior Life, Roslindale, Massachusetts, United States; John A. Burns School of Medicine University of Hawaii, Honolulu, Hawaii, United States; Hebrew Senior Life, Roslindale, Massachusetts, United States; Hebrew Senior Life, Roslindale, Massachusetts, United States; Hebrew Senior Life, Roslindale, Massachusetts, United States; Hebrew Senior Life, Roslindale, Massachusetts, United States; Hebrew Senior Life, Boston, Massachusetts, United States

## Abstract

Several validated scales have been developed to measure frailty, yet it remains unknown how these measures are related. We used data from 7,070 community-dwelling older adults who participated in National Health and Aging Trend Study round 5 to construct a crosswalk among frailty measures. We operationalized the 60-item Frailty Index (FI), Study of Osteoporotic Fracture (SOF) Index, FRAIL Scale, Frailty Phenotype, Clinical Frailty Scale (CFS), Vulnerable Elder Survey-13 (VES-13), Tilburg Frailty Indicator (TFI), Groningen Frailty Indicator (GFI), and Edmonton Frailty Scale (EFS). Missing data, needed for the calculation of frailty scores, were imputed using multiple imputation by chained equations method. We then linked the scores of each frailty measure to FI using the equipercentile method, a statistical procedure that links different scales by equating percentile distributions. Participants considered frail on FI (cutpoint of 0.25) corresponded to the following scores on each frailty measure: SOF 1.3, FRAIL 1.7, Phenotype 1.7, CFS 5.3, VES-13 5.5, TFI 4.4, GFI 4.4, and EFS 5.8. Conversely, individuals considered frail on each frailty measure corresponded to the following FI scores: 0.37 (SOF), 0.40 (FRAIL), 0.42 (Phenotype), 0.21 (CFS), 0.19 (VES-13), 0.28 (TFI), 0.22 (GFI), and 0.37 (EFS). The CFS, VES-13, TFI and GFI each discriminates between non-frail and frail people in the pre- to mildly frail spectrum on the FI, whereas the SOF, FRAIL Scale, Phenotype, and EFS detect those in the higher frailty spectrum on the FI. Our results provide clinicians and researchers with a useful tool to convert and interpret frailty across scales.

